# Completeness of Medical Records of Trauma Patients Admitted to the Emergency Unit of a University Hospital, Upper Egypt

**DOI:** 10.3390/ijerph18010083

**Published:** 2020-12-24

**Authors:** Zeinab Mohammed, Ahmed Arafa, Shaimaa Senosy, El-Morsy Ahmed El-Morsy, Emad El-Bana, Yaseen Saleh, Jon Mark Hirshon

**Affiliations:** 1 Public Health and Community Medicine Department, Faculty of Medicine, Beni-Suef University, Beni-Suef 62521, Egypt; zynab.mohammed@med.bsu.edu.eg (Z.M.); Ahmed011172@med.bsu.edu.eg (A.A.); shoshoahmed80@yahoo.com (S.S.); Elmorsy50@yahoo.com (E.-M.A.E.-M.); 2 Department of Emergency Medicine, University of Maryland School of Medicine, Baltimore, MD 21201, USA; jhirshon@umaryland.edu; 3 Department of Public Health, Graduate School of Medicine, Osaka University, Osaka 565-0871, Japan; 4 Department of Orthopedic Surgery, Faculty of Medicine, Beni-Suef University, Beni-Suef 62521, Egypt; elbanaemad@yahoo.com; 5 College of Medicine, University of Illinois at Chicago, Chicago, IL 60607, USA; 6 Department of Epidemiology and Public Health, University of Maryland School of Medicine, Baltimore, MD 21201, USA

**Keywords:** trauma registries, trauma, medical records, emergency departments, LMICs, Egypt

## Abstract

Trauma records in Egyptian hospitals are widely suspected to be inadequate for developing a practical and useful trauma registry, which is critical for informing both primary and secondary prevention. We reviewed archived paper records of trauma patients admitted to the Beni-Suef University Hospital in Upper Egypt for completeness in four domains: demographic data including contact information, administrative data tracking patients from admission to discharge, clinical data including vital signs and Glasgow Coma Scale scores, and data describing the causal traumatic event (mechanism of injury, activity at the time of injury, and location/setting). The majority of the 539 medical records included in the study had significant deficiencies in the four reviewed domains. Overall, 74.3% of demographic fields, 66.5% of administrative fields, 55.0% of clinical fields, and just 19.9% of fields detailing the causal event were found to be completed. Critically, oxygen saturation, arrival time, and contact information were reported in only 7.6%, 25.8%, and 43.6% of the records, respectively. Less than a fourth of the records provided any details about the cause of trauma. Accordingly, the current, paper-based medical record system at Beni-Suef University Hospital is insufficient for the development of a practical trauma registry. More efforts are needed to develop efficient and comprehensive documentation of trauma data in order to inform and improve patient care.

## 1. Introduction

Globally, trauma accounts for more than 10% of mortality, resulting in around five million deaths annually [1]. Trauma is considered a leading cause of death and disability, disproportionately affecting lower- and middle-income countries (LMICs), where 90% of the global trauma burden occurs [2]. A critical step in improving trauma care is to establish and evaluate the local epidemiology of injuries. Understanding common mechanisms of injury and presentations specific to a particular population enables healthcare and public health systems to take proactive steps in both primary prevention (by reducing risks of injury) and secondary prevention (by improving quality of care). Accordingly, the existing literature has demonstrated that meticulous record-keeping strongly correlates with quality of care, while deficient record-keeping is often an indicator of poor quality of care [3].

In the past, the lack of available medical records in LMICs has made estimating the actual trauma burden challenging [4]. However, trauma documentation has recently started to receive more attention in LMICs as a crucial mechanism of data collection, allowing hospitals to audit and improve care. This process has been restrained by the lack of resources available to efficiently establish a quality trauma registry, hindering injury management in such countries [5].

In Egypt, the trauma burden is a hidden epidemic. The extensive (and likely expanding) problem of traumatic injury is understated due to the lack of proper documentation and the absence of data available at the national level. This negatively affects the quality of care given to trauma patients, with an exaggerated effect on regions in Upper Egypt due to the lack of human and financial resources available in healthcare facilities [6]. Upper (or Southern) Egypt is the region comprising all the riverside territory south of the Nile delta. It consists of nine governorates, which are among the least privileged governorates in Egypt in terms of average income, educational attainment, and funding [7]. More than 120 km south of Cairo, the studied hospital is located in the governorate of Beni-Suef, an area that is representative of conditions seen across Upper Egypt.

Despite the growing international consensus on the importance of trauma registries and medical record systems, Beni-Suef University Hospital, like most other tertiary-care hospitals in Egypt, lacks an electronic medical record and relies on paper-based recordkeeping. The responsibilities of data collection and data entry usually fall to the individual nurse’s or physician’s preference, as there is no assigned clerk nor systemized protocol for this task. Furthermore, ambulatory and police records are not routinely collected nor are they usually available or reliable. In the event of death, post-mortem analysis is usually rejected by the patient’s relatives due to cultural norms. Therefore, despite their hypothesized insufficiency, in-hospital records are largely the only local data sources currently available that could potentially be used to inform trauma care. In this study, we investigated the medical records of admitted trauma patients for the completeness of several pre-identified variables in order to evaluate the adequacy of the current medical record as a basis for the development of future trauma registries in hospitals in Upper Egypt.

## 2. Materials and Methods

### 2.1. Study Design

This study was a retrospective, cross-sectional analysis of the medical records of all trauma patients admitted to Beni-Suef University Hospital over the eight months from 1 January to 31 August 2016. The length of the study period was limited to roughly two academic semesters due to the transport of hospital archives to outside storage locations, making them inaccessible to the authors. However, the chosen study period approximates an academic year, including 2–3 months from each of the three traditional seasons of Egyptian life (i.e., the flooding, growing, and harvest seasons), allowing for an adequate and representative sample of the year.

### 2.2. Setting

Beni-Suef University Hospital is an urban, tertiary-care hospital and the third-largest university hospital in Upper Egypt. It is located at the junction point of Upper and Lower Egypt and serves approximately 3.1 million inhabitants, comprising a diverse mixture of backgrounds (in terms of income level, educational attainment, urban vs. rural etc.) common to the region. The hospital has 423 beds and 8 intensive care units. It has an emergency unit that receives all medical and surgical emergencies for both adult and pediatric populations. Furthermore, it is the only hospital in the governorate that is equipped and staffed to provide care for patients with severe trauma. Notably, the hospital emergency unit uses entirely paper charts. All patient data are handwritten onto standardized forms, which are then archived following patient discharge.

### 2.3. Participants and Data Sources

All the medical records of patients who presented to the Emergency Unit of Beni-Suef University Hospital from 1 January to 31 August 2016 were reviewed to identify patients who could be potentially included in a trauma registry. Patients meeting at least one of the following criteria were included: (1) inpatient admissions (>24 h) due to trauma (including burns patients), (2) transferred trauma patients from other local hospitals, (3) deaths attributable to trauma, or (4) patients requiring trauma team activation/consult. Atraumatic medical admissions were excluded in addition to medical records that were damaged or illegible. Trauma cases were differentiated from medical cases on the basis of clinical data such as the primary diagnosis required upon admission. Data extraction was performed manually from the paper medical archives, with each case being recorded on a structured form (Document S1). These forms contained a set of information fields (such as “Age,” “Address,” “Pulse,” etc.) that were used as variables for this study. Only one of the studied variables did not have a designated field in the medical record forms, which was the case’s eligibility for a trauma consultation. To identify if the case resulted in a trauma consultation, we checked the field in the chart that is reserved exclusively for comments from the trauma team. A filled section would indicate that a trauma consultation was done.

### 2.4. Study Variables

A computerized data collection form was created, and the data for each record were entered directly into the program. Data from the following four variable domains were collected: (1) patient demographic/contact information (age, gender, residence, occupation, and contact information); (2) administrative data regarding patient admission and course (date/time of arrival to the hospital, mode of arrival, date/time of discharge, status on discharge, presence of a hospital identification number, and name/signature of the healthcare provider who admitted the patient); (3) clinical data, specifically vital signs (blood pressure, heart rate, respiratory rate, temperature, and SpO2) and the patient’s Glasgow Coma Scale (GCS) score; and (4) data describing the injury incident (mechanism of injury, activity at the time of injury, and site/location of the incident). Additionally, if a trauma team activation occurred, two more data fields were collected: the time of activation of the trauma team and the time of arrival of the trauma team. We defined completeness of data as the extent to which the cases contained the above-defined variables, as has been done elsewhere [8]. To measure completeness for a given record, completed fields were given one point while missing fields were given zero points. The composite completion rate of each of the investigated four domains was then calculated, and the completeness of each domain was further categorized into one of the following groups: low (<50% completion rate), intermediate (50–75% completion rate), and high (>75% completion rate).

### 2.5. Ethical Approval

Approval for this study was obtained from the Research Ethics Committee of the Faculty of Medicine at Beni-Suef University and from the Institutional Review Board at the University of Maryland in Baltimore.

## 3. Results

**Figure 1:**
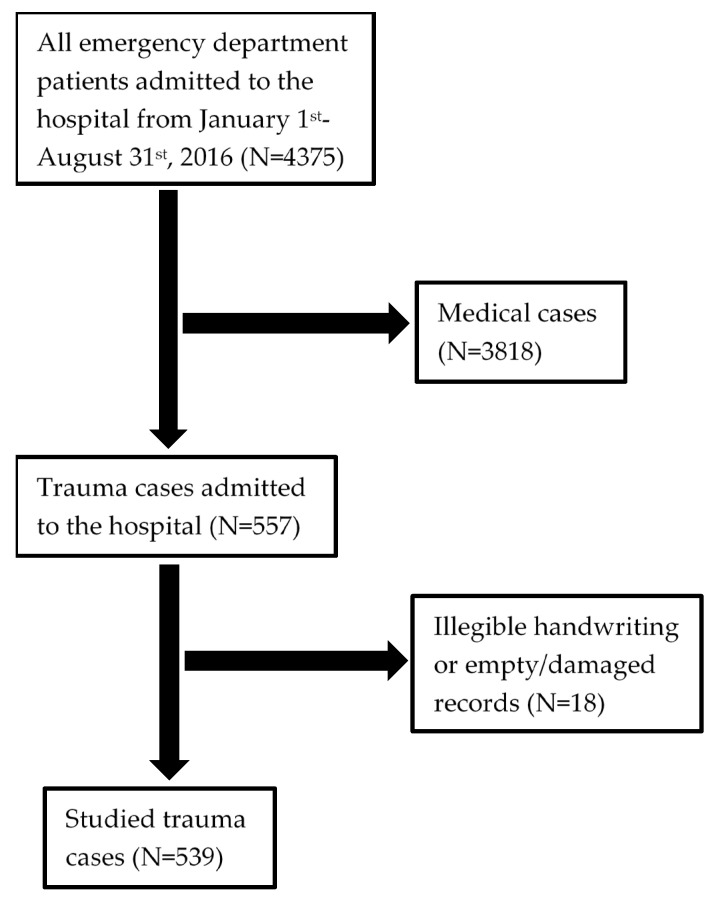
Flow diagram of included and excluded records for analysis.

A total of 557 trauma cases were admitted to the hospital during the study period, representing 12.7% of the total emergency admissions to Beni-Suef University Hospital. Out of these 557 records, 18 were damaged or illegible. These records were subsequently excluded from further analysis, leaving 539 patient records (Figure 1).

**Figure 2:**
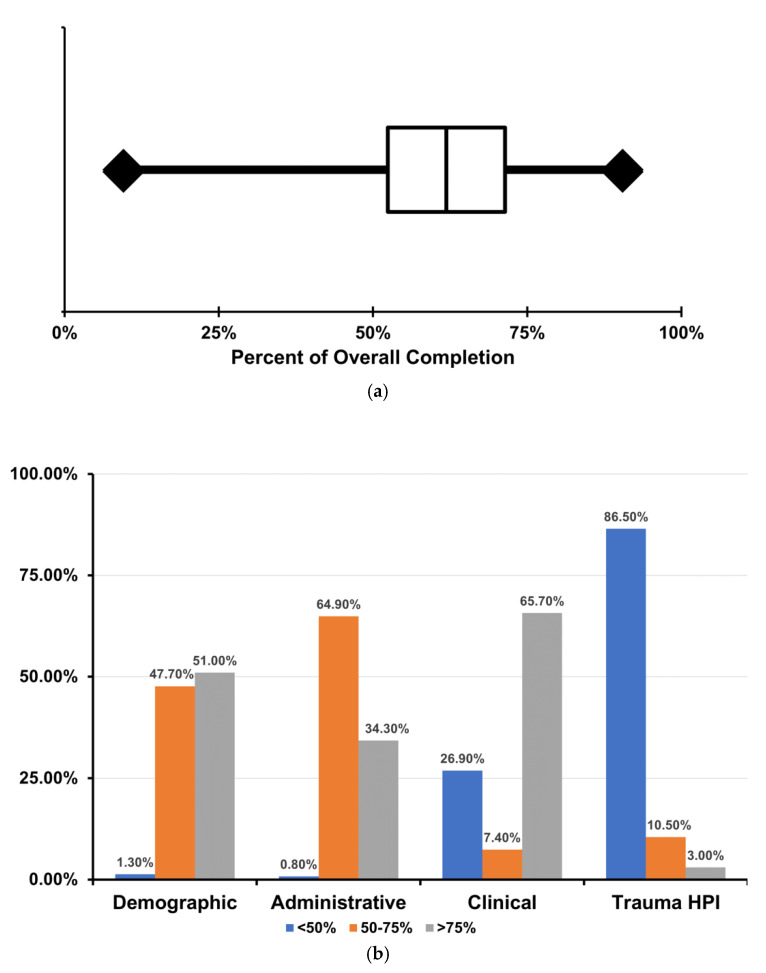
(**a**) Box plot of composite completion rates for the 539 included records. (**b**) Proportion of records with low (<50%), intermediate (50–75%), or high (>75%) levels of completion for each data category. Trauma HPI refers to the history of present illness. This category corresponds to the domain comprising all the data fields describing the cause of trauma and inciting incident.

The results revealed significant deficiencies in overall data completion rates. The median completion rate was 61.9% of the measured data fields, with the central two quartiles of records ranging from 52.4% to 71.4% completion (Figure 2a). Breaking down completion rates into the respective four studied domains highlighted particular areas of deficiency. For example, while close to half (51%) of the records had more than 75% of patient demographic fields completed, only 34.3% of the records achieved the same completion rates of the required administrative fields. The overwhelming majority of the records (86.5%) contained less than 50% of the requested information describing the causal incident (Figure 2b). No significant differences were found between completion rates of daytime and overnight admissions for those records with recorded admission times. However, it should be noted that the time of arrival was only documented in 25.8% of the records, leading to a skewed sample with likely insufficient power to detect such a difference if present.

### 3.1. Completion of Demographic Fields

Delving specifically into the demographics domain, the most complete data fields were age, sex, and residence. These were present in 97.0%, 98.3%, and 97.8% of the records, respectively. These fields are generally recorded by the clerical and nursing staff upon admission for the patient to officially enter the hospital system, likely explaining their high rates of completion. Conversely, phone numbers or other contact information, which are critical to enabling patient follow-up, were available in only 43.6% of the cases. Occupation (for the 380 adult patients) was also rarely recorded (18.4% of records). The overall completeness of demographic fields stood at 74.3% (Table 1).

### 3.2. Completion of Administrative Fields

Concerning the administrative domain, the time of arrival (a critical determinant for the care of unstable patients) was present in only 25.8% of cases, while the mode of arrival was present in 38.3% of cases. The hospital ID number as well as admitting physician were regularly present (88.9% and 82.0% of cases, respectively). Finally, for the 52.3% of cases that required trauma team activation, the time of activation was never recorded, and the time of the team’s arrival was recorded in only a rare 6.4% of cases. The overall completeness of administrative fields stood at 66.5% (Table 2).

### 3.3. Completion of Clinical Fields

Completion of clinical fields was variable. In terms of vitals, records contained the patient’s blood pressure in 78.5% of cases, pulse in 67.5%, respiratory rate in 70.9%, temperature in 22.1%, and SpO2 in just 7.6%. The patient’s GCS score was present in 83.7% of cases. The overall completeness of clinical fields stood at a striking 55.0%, though this was mostly attributable to the absence of SpO2/temperature data (Table 3).

### 3.4. Completion of Fields Detailing the Cause of Injury

The final domain, comprised of data describing the causal injury event, was the least complete category. The mechanism of injury was documented in 11.9% of the cases, the activity at the time of injury in 21.7%, and the site/location of the inciting event in 22.8%. Overall, only 19.9% of fields in this domain were completed, indicating a stark deficiency in a category critical for epidemiological surveys (Table 4).

## 4. Discussion

This study revealed significant deficiencies in the documentation of all types of patient data within the trauma records archived at Beni-Suef University Hospital. After analysis, trauma patients were found to compose 12.8% of all emergency unit admissions over the study period, roughly 70 patients a month. However, the poor quality of the archives identified in this study casts doubt as to whether all patient admissions were actually being documented, potentially masking the real trauma burden within the Beni-Suef governorate. These deficiencies additionally hinder the development of risk-prevention strategies and ultimately undermine the quality of the provided healthcare.

The issue of deficient medical records revealed in this study is similar to that reported in other LMICs, reinforcing the importance of continuing to improve data collection in resource-constrained environments. The incompleteness of medical records in many LMICs has been studied and shown elsewhere. For example, a prospective analysis of the trauma registry in a regional hospital in Cameroon showed missing details in the demographic, administrative, and clinical data of 5617 patients [9]. However, the reported deficiencies were less frequent than those detected in our study, revealing a particular inadequacy in Upper Egypt. Similarly, analysis after the creation of a multicenter trauma registry in India showed higher rates of completion for all parameters compared to the current study, highlighting the need for local interventions [10].

Inadequacy of medical records is a problem not just limited to LMICs and has been reported in some high-income countries as well. In the Netherlands, de Mul and Berg reported incompleteness of trauma records that was significant enough to affect quality assessment [3]. The authors suggested appointing a trained clerk, who would be a nurse or a physician specifically tasked with the records system. Similarly, Weiskopf et al. assessed the completeness of records in a non-profit hospital in New York City and concluded that only half of the records could be considered complete [11]. However, the authors also reported significant improvement at the hospital over the period from 1986 to 2012. While medical record-keeping has likely improved in Egypt, the current status, as shown in this study, still lags far behind, significantly hampering clinical care.

Consistent with this, our study showed absences in crucial data that directly affect the management of patients, including vital signs and the history of the presenting injury. It is of note that SpO2 was recorded (and thus likely only measured) in just 7.6% of patient cases, which is particularly concerning considering the importance of oxygenation status to the evaluation of trauma patients. The absence of such data can prevent appropriate evaluation, management, and follow-up in a time-sensitive and high-stakes environment.

Moreover, this study identified a concerning absence of data that are particularly essential for prevention strategies. To begin with, the above data analysis underreports the deficiencies in the record system, as 18 records were excluded due to being completely illegible or damaged. This in itself is concerning, as it means that the medical charts of some patients are completely inaccessible. With respect to primary prevention, information describing the causal traumatic incident was recorded in less than 25% of records for every field in that domain. Collectively, only 19.9% of fields in that domain were completed. Accordingly, it would be unfeasible with the current availability of data to establish a reliable epidemiological study to identify health risks that could be reduced through public health interventions. With respect to secondary prevention, important logistical information such as time of arrival, time of trauma team activation, and time of trauma team arrival were rarely recorded. These are critical facets both for the care of unstable patients and for internal quality assessments. Furthermore, the patient’s contact information was recorded for less than half of the patients, making it additionally unfeasible for the hospital to follow up with patients after discharge if needed (or to contact them in virtually any way). The patient’s residence, though almost universally completed, is unfortunately not a reliable type of contact information in Egypt, as many areas lack unit numbers or street names. Consequently, the patient may report a village or neighborhood that hosts thousands of people as their residence.

Although the managerial system and the medical staff are primarily responsible for the incomplete records, other factors related to the patient’s specific trauma incident may further complicate documentation. For instance, the severity of trauma and the nature of emergent pre-hospital care may interfere with obtaining complete documentation [12]. Details such as the mechanism of injury and the activity of patients at the time of injury may be missing in the ambulance report, if available [13]. Furthermore, the patient in many situations due to their condition might not be able to provide the required details [14].

While detecting the magnitude of trauma is beyond the aim of this study, we can reasonably conclude that trauma is a sizable problem in Upper Egypt. Mahran et al. assessed trauma records at Assiut University Hospital between 2002 and 2009 and reported a significant rise in the proportion of trauma admissions from 9.3% to 15.3% of all admissions [6]. Similar rises in trauma incidence were reported in high-income countries such as the USA [15] and the UK [16] as well as LMICs like Cameroon [9] and Ethiopia [17]. Accordingly, substantial work is needed to meet the reported inadequacy of documentation in order to help inform and ameliorate the expanding impact of trauma internationally.

Detecting the deficiencies in trauma documentation at Beni-Suef University Hospital is just the first step in a process with the ultimate goal of improving clinical care and injury prevention in the area. Work has already been completed assessing the attitudes, goals, and suggestions of hospital faculty and staff towards the improvement of trauma documentation. The results of this study are pending. The next stage of the project would be the implementation of a trauma registry and interventions to improve the quality and completeness of documentation. One implementable action that could have a modest improvement in record-keeping could be to train nurses and physicians on the importance of maintaining complete records for trauma patients. Additionally, appointing a designated trauma clerk to be responsible for real-time documentation could significantly improve data collection in the absence of an electronic record system which would streamline this process. Feasibility in light of the university’s resources, however, needs to be assessed first. Following the trial of such interventions, we can reassess the results of the current study after 2–3 years to measure quality improvement. Ultimately, we wish to establish a robust system of data collection that will inform primary and secondary prevention and improve clinical care.

### Limitations

Overall, this study highlighted the need for a well-developed trauma registry to rectify the significant defects in the current system of documentation at Beni-Suef University Hospital. Yet, this study had some limitations that should be taken into consideration. Firstly, the retrospective nature of the study does not allow for real-time evaluation of the accuracy of the data and theoretically may also not be reflective of the current status of the hospital given a few years have passed since the study period. That being said, this is unlikely given the lack of major initiatives or changes to data collection in the interim. Secondly, this study was done at a single institution in Upper Egypt, and thus we were unable to compare the results against results from other hospitals in the country. Accordingly, the findings of this study may be generalizable to other similar university hospitals in the region, but not necessarily to public hospitals, where medical documentation is likely even worse since academic pressures generally contribute to an improvement in data collection. It may also not be comparable to hospitals in the city centers (such as Cairo or Alexandria) of Lower Egypt, where documentation may be impacted by renovated electronic systems or better funding.

Furthermore, compared to other studies that adopted more sophisticated definitions for incompleteness that evaluated the quality of recorded information [11,18,19], we used a more simplistic definition, focusing on the presence or absence of data fields. Such information has never been characterized in the region and required the establishment of a baseline, despite limited resources available. A confounding issue with this type of study is that the presence of data in the patient’s record may be skewed by the acuity and severity of the patient’s presentation. Accordingly, the lack of data may not necessarily be reflective of the quality or comprehensiveness of care, though it still prevents the creation of an effective registry. The impact of the severity and acuity of the trauma on record completion is particularly important as it would inform solutions to aid in creating a registry. For instance, if record completion suffers due to clinicians being occupied by injuries in need of critical care, then the appointing of a designated trauma clerk without clinical duties would be an effective means of improving these records. This impact can be assessed in a future study by correlating completion levels to severity of trauma determined by a scale like the Injury Severity Score as well as by making comparisons to completion rates for atraumatic medical admissions.

An additional confounding issue is that disease trends in different seasons could impact the completion of documentation. As stated prior, the study period included samples from each of the three traditional seasons in the Egyptian year. Consequently, it would be unexpected for the results to change significantly with a longer study period, since changes in weather and activity patterns were covered adequately. However, a longer study would be needed for formal comparison of each season and localization of deficits to particular seasonal trends. Finally, we could not evaluate the accuracy of data by comparing the hospital records to other records such as police records or ambulance records because these are not usually available in Egypt.

## 5. Conclusions

In conclusion, this study assessed the completeness of trauma records at Beni-Suef University Hospital, highlighting significant deficiencies in these records. Work needs to be done to improve documentation, so that the hospital can move forward in improving injury prevention and care. We believe one implementable action that could have a modest improvement in record-keeping could be to train nurses and physicians on the importance of maintaining complete records for trauma patients. Additionally, appointing a clerk to be responsible for the record system may also be of significant benefit. However, more analysis needs to be done to assess feasibility at the hospital. The ultimate goal is to establish a robust system of data collection that will inform primary and secondary prevention and improve clinical care.

## Figures and Tables

**Table 1:** Availability of demographic information in the medical records of trauma patients.

Studied Variable	Records with Completed Information *N* (% of Total) Total = 539
Age	523 (97.0%)
Sex	530 (98.3%)
Residence	527 (97.8%)
Occupation (for adults only, *N* = 380)	70 (18.4%)
Phone number or other contact information	235 (43.6%)
Completed demographic fields (Total = 2536)	1885 (74.3%)

**Table 2:** Availability of administrative data in the medical records of trauma patients.

Studied Item	Records with Completed Information *N* (% of Total) Total = 539
Date of arrival at the hospital	533 (98.9%)
Time of arrival at the hospital	139 (25.8%)
Mode of arrival to the hospital	206 (38.2%)
Date of discharge	538 (99.8)
Status at discharge	532 (98.7)
Hospital ID number	479 (88.9)
Name/signature of admitting physician	442 (82.0%)
Time of activation of trauma team (*n* = 282)	0 (0%)
Time of arrival of trauma team (*n* = 282)	18 (6.4%)
Completed administrative fields (Total = 4337)	2869 (66.5%)

**Table 3:** Availability of clinical data in the medical records of trauma patients.

Studied Item	Records with Completed Information *N* (% of Total) Total = 539
Systolic blood pressure	423 (78.5%)
Pulse	364 (67.5%)
Respiratory rate	382 (70.9)
SpO2	41 (7.6%)
Temperature	119 (22.1%)
Glasgow Coma Scale	451 (83.7%)
Completed clinical fields (Total = 3234)	1780 (55.0)

**Table 4:** Availability of data describing the injury incident in the medical records of trauma patients.

Studied Item	Records with Completed Information *N* (% of Total) Total = 539
Mechanism of injury	82 (15.2%)
Activity at time of injury	117 (21.7%)
Site/location of incident	123 (22.8%)
Completed injury data fields (Total = 1617)	322 (19.9%)

## Data Availability

The majority of the data used in this study is present in the article above. Source data available from the authors upon request due to university policies.

## Supplementary Materials

The following are available online at https://www.mdpi.com/1660-4601/18/1/83/s1, Document S1: Data Collection Pro-Forma.
