# Supplementary material for: Completeness of Medical Records of Trauma Patients Admitted to the Emergency Unit of a University Hospital, Upper Egypt
Source: Int J Environ Res Public Health. 2020 Dec 24;18(1):83. doi: 10.3390/ijerph18010083 (PMC7795587; doi:10.3390/ijerph18010083)
Supplement: Supplementary file 1 [file ijerph-18-00083-s001.pdf]

Data Sheet Collection Form

Section 1: Demographic data

1- Age (years) …………………………….
2- Sex 1-Male 2-Female
3- Residence …………………………….
4- Occupation 1-Present 2-Absent
5- Phone number 1-Present 2-Absent
6- Other contact information (e.g. relative's phone number, ID number, etc.) 1-Present 2-Absent

Section 2: Administrative data: this section showed all administrative data that should be collected from every individual patient presenting to the emergency unit:

1- Time of arrival to the hospital 1-Present 2-Absent
2- If present, what time: …………………………….
3- Date of arrival to the hospital 1-Present 2-Absent
4- Mode of arrival to the hospital 1-Present 2-Absent
5- Date of Discharge 1-Present 2-Absent
6- Status at Discharge 1-Present 2-Absent
7- Hospital ID given 1- Yes 2- No
8- Admitting physician signature 1-Present 2-Absent
9- Patient status required activation of trauma team 1-Yes 2-No
10- If yes; the time of activation is present 1-Present 2-Absent
11- The time of arrival of trauma team 1-Present 2-Absent

Section 3: Availability of clinical data in the medical records at admission:

1- Vital signs
a) Systolic blood pressure 1-Present 2-Absent
b) Pulse 1-Present 2-Absent
c) Respiratory rate 1-Present 2-Absent
d) Oxygen saturation 1-Present 2-Absent
e) Temperature 1-Present 2-Absent

2- **Glasgow Coma Scale score** 1-Present 2-Absent

Section 4: Data describing the Causal Injury incident:

1- Mechanism of injury ………….
   1-Present 2-Absent
2- Activity at time of injury ………….
   1-Present 2-Absent
3- Site/ location of incident ………….
   1-Present 2-Absent

.
